# Efficacy of Navigation Systems With Smart Delivery Tools in Enhancing the Accuracy of Percutaneous Pedicle Screw Insertion

**DOI:** 10.7759/cureus.78656

**Published:** 2025-02-06

**Authors:** Takeshi Umebayashi, Yasukazu Hijikata, Takaoki Kimura, Nahoko Kikuchi, Takeshi Hara, Keiichi Tsuda, Shinji Kumamoto, Daichi Kawamura

**Affiliations:** 1 Department of Spine Surgery, Tokyo Spine Clinic, Tokyo, JPN; 2 Spine and Low Back Pain Center, Kitasuma Hospital, Kobe, JPN; 3 Department of Spine Surgery, Fukuoka Spine Clinic, Fukuoka, JPN

**Keywords:** computer-assisted surgery, fluoroscopy, minimally invasive surgical procedures, spinal fusion, surgical instruments

## Abstract

Purpose

Minimally invasive surgical techniques are advancing in spinal surgery, creating a need for the development of surgical support systems. This study evaluates the efficacy of a compact navigation system with smart delivery tools in percutaneous pedicle screw insertion.

Methods

This retrospective observational study included consecutive thoracic or lumbar spinal fusion patients with percutaneous pedicle screw placement treated from November 2022 to July 2023 in a Japanese private hospital. Primary outcomes were screw deviations (classified as any deviation, deviations of more than 2 mm, deviations from the medial to the caudal side). Pedicle screws were divided into two groups: those placed with the navigation system and those placed with traditional fluoroscopic guidance (non-navigation). Fisher's exact test and a generalized estimation equation for the prevalence ratio were conducted, adjusting for potential confounders.

Results

This study evaluated 492 pedicle screws (190 in the navigation group) in 78 patients. The median age was 70.5 years, and the most common condition was foraminal stenosis (26 patients, 33%). Of the study participants, 40 (51%) were male. Any deviation, deviations of more than 2 mm, and deviations from the medial to the caudal side were observed in 16 screws (8.4%) vs. 54 screws (21%) (p<0.001), six screws (3.2%) vs. 31 screws (12%) (p<0.001), and five screws (2.6%) vs. 10 screws (3.8%) (p=0.5) in the navigation and non-navigation groups, respectively. The adjusted prevalence ratios of the navigation group for any deviation, deviations of more than 2 mm, and deviations from the medial to the caudal side were 0.51 (95%CI 0.27, 0.98), 0.33 (95%CI 0.14, 0.78), and 0.88 (95%CI 0.26, 3.05), respectively.

Conclusion

This study suggests that compact navigation systems with smart delivery tools may improve screw placement accuracy in spinal surgeries.

## Introduction

Minimally invasive techniques are rapidly advancing in the field of spine surgery, providing significant benefits to patients such as reduced postoperative pain and shorter recovery times [1]. However, these techniques come with the trade-off of increased surgical complications due to the difficulty in visualizing anatomical structures. Therefore, the development of surgical support equipment, such as navigation systems, is crucial to perform the procedures more safely [2]. Minimally invasive surgical procedures also require more frequent X-ray confirmation of anatomical structures. In recent years, healthcare professionals have renewed their focus on radiation-related adverse events [3,4], highlighting the need for navigation systems that can reduce radiation exposure.

Advances in computed tomography (CT)-based spinal surgical navigation systems have been reported to improve the accuracy and safety of pedicle screw placement [5-7]. These conventional CT-based navigation systems fix a tracking ball to the patient’s body and use a compound camera outside the surgical field to recognize the position of the tracking ball. The tracking ball attached to instruments such as screwdrivers and needles is then recognized, projecting the instruments onto the pre-configured three-dimensional (3D) field. This setup requires a large space with no obstacles between the patient's body, the instruments, and the compound camera.

A compact navigation system with smart delivery tools uses a different tracking system to recognize 3D fields [8]. A monocular camera attached to the instruments recognizes luminescent targets and calculates their relative positions, thus eliminating the need for a large space other than the space for screw insertion. If this novel system is as effective in ensuring screw insertion accuracy as conventional navigation systems, it offers the advantage of requiring less operating space. However, although a small number of cases have been reported [9], the effectiveness of this system is still unclear.

The purpose of this study was to evaluate the efficacy of the compact navigation system with smart delivery tools in percutaneous pedicle screw insertion by comparing it with fluoroscopically guided insertion.

## Materials and methods

Study design and participants

This cross-sectional study retrospectively extracted data from the medical records of Tokyo Spine Clinic, Tokyo, Japan, covering the period from November 2022 to July 2023. The study was conducted in accordance with the Declaration of Helsinki [10] and the Strengthening the Reporting of Observational Studies in Epidemiology (STROBE) Statement [11] and was approved by the Institutional Review Board of the Institute for Health Outcomes and Process Evaluation Research (approval number: 202401). Consecutive cases where pedicle screws were placed in the thoracic or lumbar spine were included. Cases involving only screw replacement and staged surgery were excluded from the study. All data were de-identified to ensure confidentiality and an opt-out policy was implemented, eliminating the need for individual consent.

Interventions

**Figure 1:**
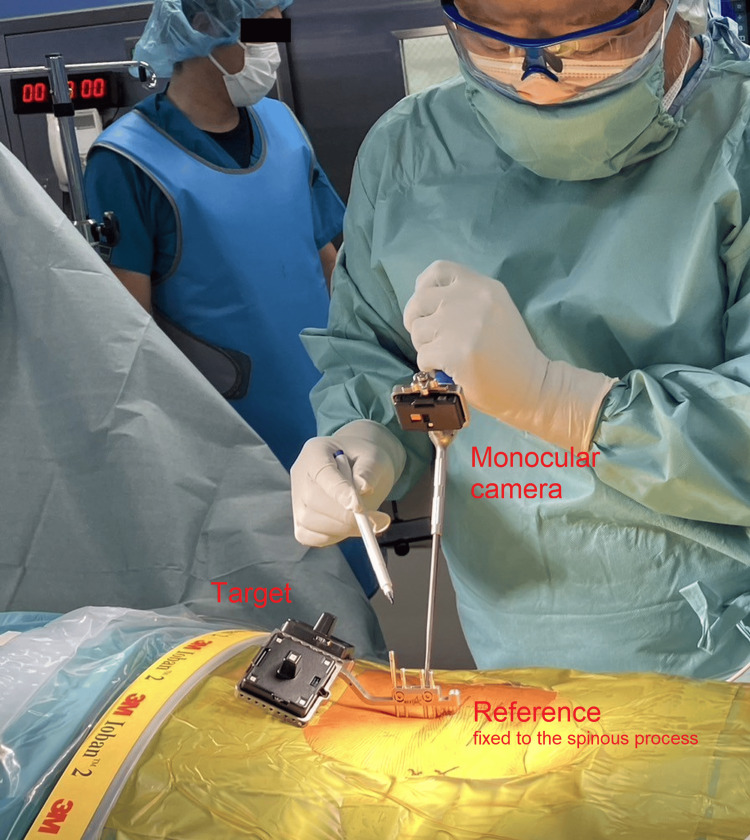
Intra-operative photographs of the navigation system in use The target is mounted on the reference fixed to the spinous process, and the monocular camera attached to the probe recognizes the target, transmitting positional information via Bluetooth to the control unit. Dr. Takeshi Umebayashi is holding the probe with the attached monocular camera. The surgeon can perform the procedure while confirming the real-time position of the probe on the 3D field displayed on the control unit, without the need for fluoroscopy. Conventional navigation systems rely on tracking balls attached to both the reference and the probe, which are recognized by an external camera outside the surgical field to determine positional information. In contrast, this system completes positional tracking through direct interaction between the reference and the probe. At this stage, intraoperative CT imaging has already been completed, and the fiducial has been removed.

The center where the study was conducted acquired a state-of-the-art navigation system, NextAR TS (Tracking System) (Medacta International, Castel San Pietro, Switzerland) in July 2022, which was used during the placement of pedicle screws. The allocation of navigation system use was based on surgeon preference or operating room availability and was not randomized. When the navigation system was not used, pedicle screw placement was performed with the assistance of a standard fluoroscopy device. All pedicle screws were placed percutaneously. Intra-operative photographs of the navigation system in use are shown in Figure 1.

In the navigation group, the surgeon first fixed a reference frame to the spinous process near the treated spinal level. A fiducial and a tracking target were then attached to the reference, and intraoperative CT images were acquired using the O-arm, allowing the navigation system to construct a 3D field on the control unit. After imaging, the fiducial was removed, and the surgical instrument equipped with an infrared camera was mounted onto the reference for calibration. Once calibrated, the instrument's position was projected onto the 3D field displayed on the control unit, enabling real-time visualization during pedicle screw insertion.

Outcomes

The primary outcome included any deviation of the placed screw, determined using CT coronal images, which are routinely taken within a few days after the operation. Screw deviation was assessed by examining CT coronal images to determine whether any part of the screw had breached the outermost cortical bone of the pedicle. This assessment followed the methodology of previous studies [12], and was conducted by YH, an independent evaluator not affiliated full-time with the clinic, who was blinded relative to the use of the navigation system. Secondary outcomes included deviations of more than 2 mm. Additionally, screw deviation was classified based on the direction of breach-medial, lateral, cranial, or caudal relative to the pedicle-and deviations from the medial to the caudal side (in proximity of the exiting nerve root).

Covariates

In addition to information on interventions and outcomes, data were collected on age, sex, body mass index, surgical indication, surgical approach, spinal level, screw diameter, minimum pedicle width, minimum pedicle cross-sectional area, CT values of the L1 vertebra, operative time, and blood loss. The surgical indication was categorized into five groups: spondylolisthesis, spinal deformity, foraminal stenosis, vertebral fracture, and others. The surgical approach was categorized into three groups: posterior or transforaminal lumbar interbody fusion, lateral interbody fusion, with or without posterior fixation, and other approaches including vertebral augmentation with pedicle screw fixation. The spinal level was divided into thoracic, upper lumbar (L1-3), lower lumbar (L4-5), and sacral. The minimum pedicle width and the minimum pedicle cross-sectional area were measured on the picture archiving and communication system (PACS) using preoperative CT. The minimum pedicle width was determined at the narrowest point of the relevant vertebral pedicle in the axial image. The minimum pedicle cross-sectional area was measured by fitting a maximized ellipse that did not extend into the cortex of the relevant pedicle in the coronal image. The CT values were measured on the adjacent vertebra if they could not be obtained from the L1 vertebra.

Statistical methods

First, background information on the study subjects was described overall and stratified by navigation and non-navigation groups. Depending on the variables, background information was divided into patient-specific summaries and pedicle-specific summaries. Continuous variables were summarised as median, interquartile range, and minimum-maximum, while categorical variables were presented as numbers and percentages. P-values were calculated using Wilcoxon’s rank-sum test for continuous variables and Fisher’s exact test for categorical variables to measure differences in characteristics between groups.

Second, Fisher’s exact test was employed to compare the screw deviations between navigation and non-navigation groups. In addition, generalized estimating equations of log-linked binomial distribution, with patient ID as a panel variable, were used to calculate the screw deviation prevalence ratio and its 95% confidence interval (CI) for the navigation group compared to the non-navigation group. L1 CT value, screw diameter, and minimum pedicle cross-sectional area were adjusted as confounding factors. Pedicle screws inserted in the sacrum, where the minimum pedicle cross-sectional area could not be measured, were excluded from the analysis. As a secondary analysis, similar analyses were performed for deviations of more than 2 mm and deviations from the medial to the caudal side. Statistical analyses were performed using Stata version 17 (2021; StataCorp LLC, College Station, Texas, United States).

## Results

This study included 492 pedicle screws in 78 patients. Background information on the study population is presented in Table 1. The median age was 70.5 years (IQR 60, 79), and 40 (51%) patients were male. The most common surgical indications were foraminal stenosis (33%) and spondylolisthesis (26%). There were some differences between the groups: the navigation group had a higher proportion of intervertebral foramen stenosis (46% vs. 22%) (p=0.039), a lower proportion of thoracic screws (0% vs. 13%), and a slightly smaller screw diameter (7.0 (IQR 7.0, 7.0) vs. 7.0 (IQR 7.0, 7.5)) (p<0.001). There was no clear group difference in the minimum pedicle cross-sectional area (62 mm² vs. 65 mm²) (p=0.29), but the minimum pedicle width was slightly larger in the navigation group (8.0 mm vs. 7.5 mm) (p=0.005). There were no obvious group differences in operative time and blood loss, but there was a trend toward a slightly shorter operative time in the navigation group (118 minutes vs. 130 minutes) (p=0.73).

**Table 1:** Background information of the study population Data are presented as n (%) or median (IQR), (minimum-maximum). Variables marked with an asterisk (*) are summarised on a per-patient basis, not a per-screw basis (total of 78 patients, with 37 in the navigation group and 41 in the non-navigation group). Variables marked with a dagger (†) exclude Sacral from the summary. PLIF: posterior lumbar interbody fusion; TLIF: transforaminal lumbar interbody fusion; LIF: lateral interbody fusion; IQR: interquartile range

	Total (N= 492)	Navigation Group (n= 190)	Non-navigation Group (n= 302)	p-value
Age* (years), median (IQR), (min-max)	70.5 (60, 79), (32-88)	70 (59, 79), (32-88)	71 (62, 78), (45-86)	1
Male sex*, n (%)	40 (51)	17 (46)	23 (56)	0.37
Body mass index* (kg/m^2^), median (IQR), (min-max)	23.7 (21.8, 26.9), (15.9-38.3)	23.8 (21.6, 27.7), (15.9-37.4)	23.6 (22.5, 26.7), (18.1-38.3)	0.89
Surgical Indication*, n (%)				0.039
Spondylolisthesis	20 (26)	9 (24)	11 (27)	
Deformity	7 (9.0)	1 (3.0)	6 (15)	
Foraminal Stenosis	26 (33)	17 (46)	9 (22)	
Fracture	4 (5.0)	0	4 (10)	
Others	21 (27)	10 (27)	11 (27)	
Surgical approach*, n (%)				<0.001
PLIF or TLIF	53 (68)	35 (95)	18 (44)	
LIF, with or without posterior fixation	20 (26)	2 (5.0)	18 (44)	
Others	5 (6.0)	0	5 (12)	
Spinal level, n (%)				<0.001
Thoracic	40 (8.1)	0	40 (13)	
Upper lumbar	102 (21)	12 (6.3)	90 (30)	
Lower lumbar	250 (51)	122 (64)	128 (42)	
Sacral	100 (20)	56 (30)	44 (15)	
Screw diameter (mm), median (IQR)	7 (7.0, 7.5)	7 (7.0, 7.0)	7 (7.0, 7.5)	<0.001
5 mm, n (%)	2 (0.4)	2 (1.1)	0	<0.001
5.5 mm, n (%)	4 (0.9)	0	4 (1.5)	
6 mm, n (%)	44 (9.7)	18 (9.5)	26 (9.8)	
6.5 mm, n (%)	24 (5.3)	0	24 (9.1)	
7 mm, n (%)	266 (59)	160 (84)	106 (40)	
7.5 mm, n (%)	98 (22)	0	98 (37)	
8 mm or more, n (%)	16 (3.3)	10 (5.3)	6 (2.3)	
Minimum pedicle cross-sectional width^†^(mm), median (IQR), (min-max)	7.7 (6.5, 8.7), (3.5-12.0)	8.0 (6.9, 8.7), (4.3-10.9)	7.5 (6.3, 8.6), (3.5-12.0)	0.005
Minimum pedicle cross-sectional area^†^ (mm^2^), median (IQR), (min-max)	63.6 (52.4, 74.5), (26.9-130.8)	62.2 (53.0, 73.4), (26.9-109.2)	64.9 (52.3, 76.9), (29.4-130.8)	0.29
L1 CT value (HU), median (IQR), (min-max)	116 (94, 161), (27-251)	114 (83, 172), (27-240)	137 (94, 161), (32-251)	0.68
Surgical time* (minutes), median (IQR), (min-max)	120 (94, 153), (40-502)	118 (103, 147), (80-221)	130 (91, 160), (40-502)	0.73
Blood loss* (ml), median (IQR), (min-max)	123 (50, 220), (20-1095)	120 (100, 200), (30-1095)	125 (50, 280), (20-840)	0.45

**Figure 2:**
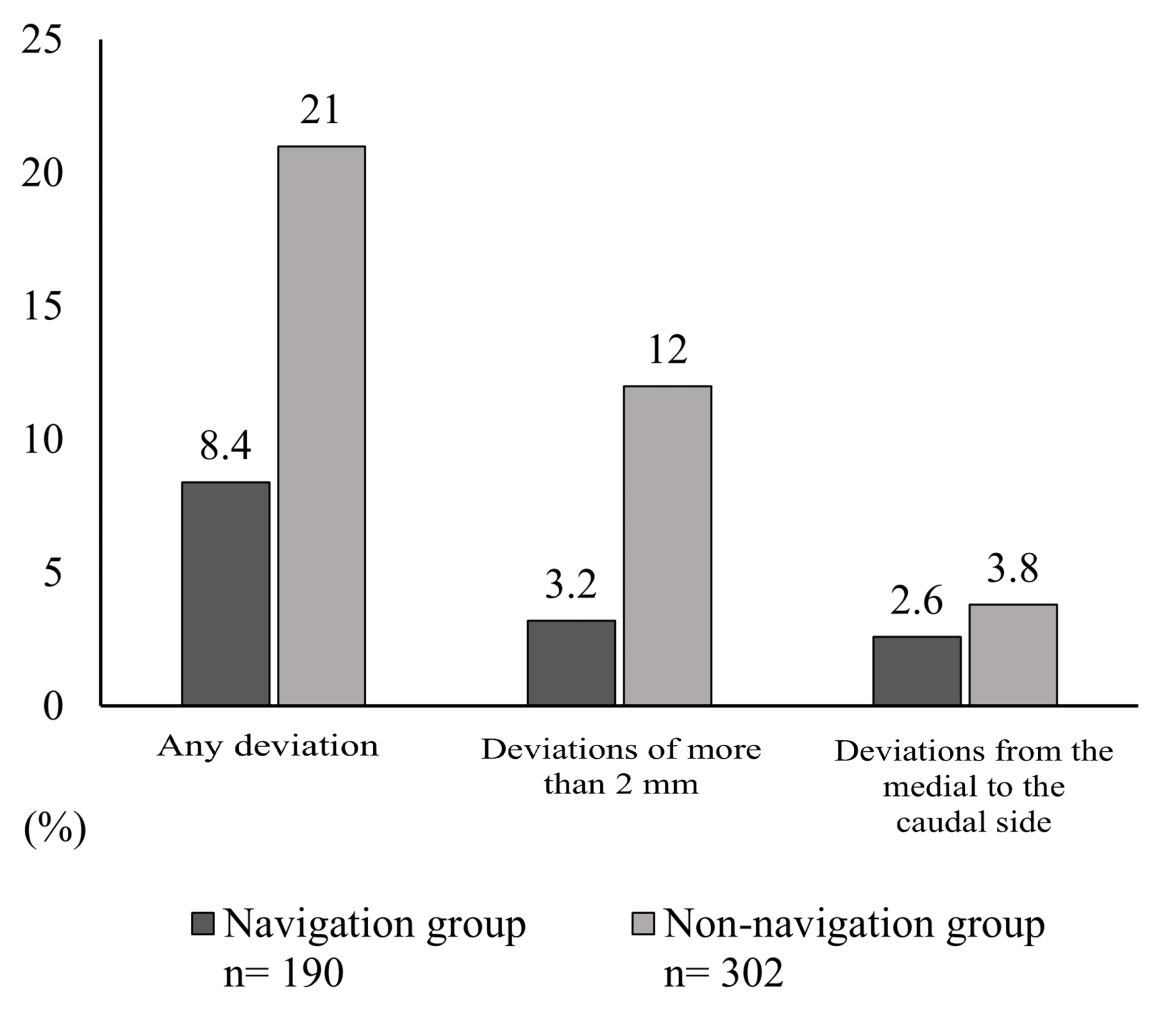
Comparison of screw deviation between the navigation and non-navigation groups Data are presented in percentage

The results for screw deviation are shown in Figure 2. There were fewer occurrences of “Any deviation” in the navigation group than in the non-navigation group (16 screws (8.4%) vs. 54 screws (21%)) (p<0.001). Similarly, there were fewer deviations of more than 2 mm in the navigation group (6 screws (3.2%) vs. 31 screws (12%)) (p<0.001). There was also a trend towards fewer deviations from the medial to the caudal side in the navigation group (5 screws (2.6%) vs. 10 screws (3.8%)) (p=0.5). Overall, deviations of any type were observed in 70 screws (15%), deviations of more than 2 mm were found in 37 screws (8.1%), and deviations from the medial to the caudal side in 15 screws (3.3%). There were no cases of nerve root irritation symptoms or re-operation.

**Figure 3:**
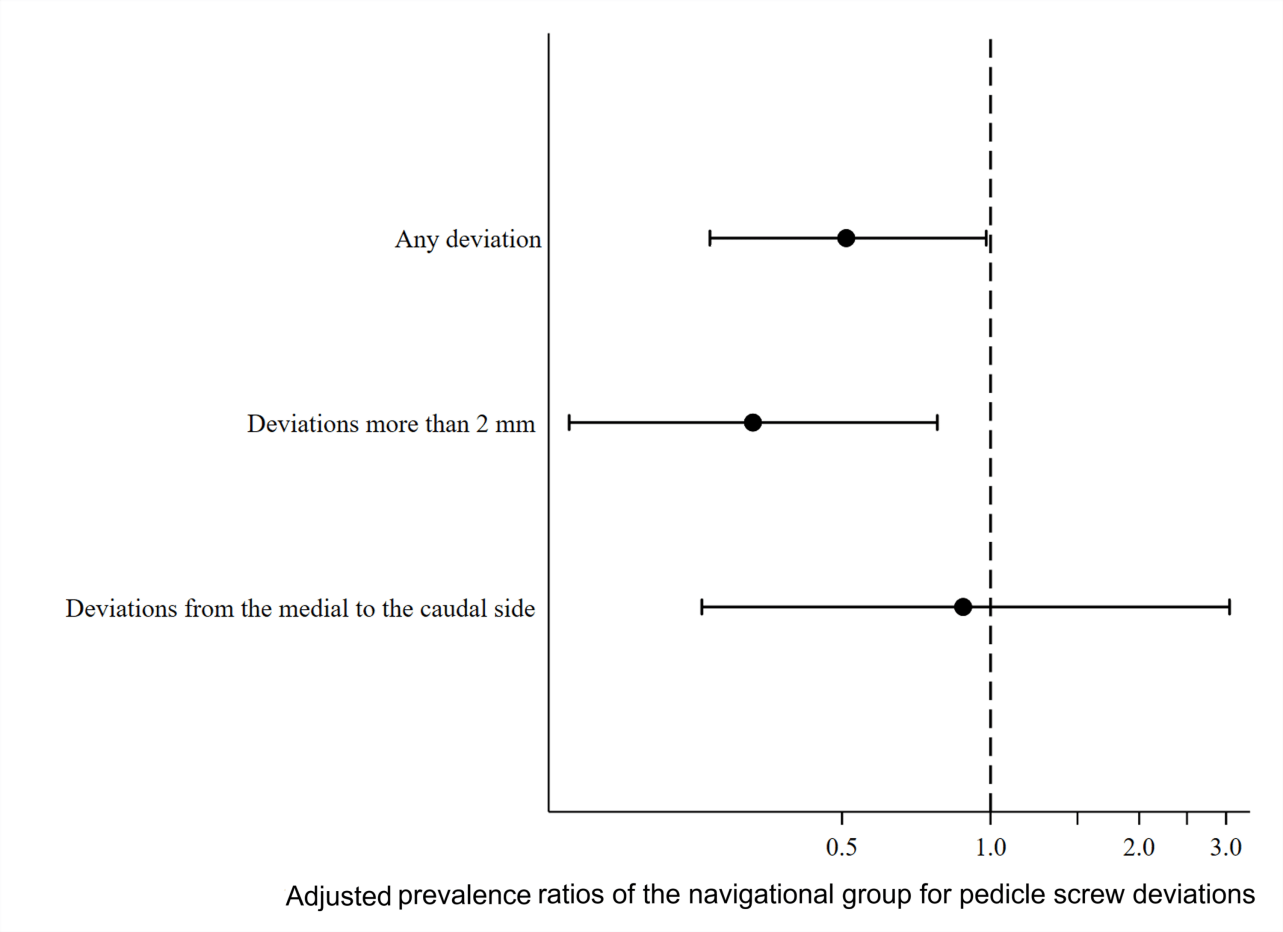
Forest plot showing adjusted prevalence ratios of screw deviations in the navigation group (N=190)

The raw and adjusted prevalence ratios for any screw deviation in the navigation group compared to the non-navigation group were 0.41 (95%CI 0.20, 0.85) and 0.51 (95%CI 0.27, 0.98), respectively (Figure 3). For deviations of more than 2 mm, the raw and adjusted prevalence ratios were 0.26 (95%CI 0.10, 0.69) and 0.33 (95%CI 0.14, 0.78), respectively. For deviations from the medial to the caudal side, the raw and adjusted prevalence ratios were 0.80 (95%CI 0.23, 2.79) and 0.88 (95%CI 0.26, 3.05), respectively.

## Discussion

This retrospective cross-sectional study, conducted at a single centre in Japan, involving 78 patients and 492 pedicle screws, demonstrated the potential efficacy of the compact navigation system with smart delivery tools in reducing screw deviations. Specifically, it was associated with approximately half of the occurrences of any deviation and about one-third of the occurrences of deviations of more than 2 mm. Further studies are needed to compare this system with conventional navigation systems, evaluate its effect on reducing total operative time, and assess its impact on the surgeon's radiation exposure to assert its overall usefulness. However, given the results of this study, the clinical utility of compact navigation systems with smart delivery tools seems promising.

This navigation system is expected to offer two main advantages over conventional systems. First, the camera attached to the instrument recognizes the target attached to the reference point that determines the 3D field and transmits only this information to the navigation unit. This linear and unidirectional exchange of positional information simplifies the calculation of the position. As a result, improved response speed and accuracy can be expected. A paper reviewing the evidence up to 2010 reported that the percentage of screws fully contained in the pedicle using navigation systems ranged from 88% to 100% (i.e., a deviation rate of 0-12%) [13]. Recent reports have indicated screw insertion accuracy of 97.4-97.8% (i.e., a deviation proportion of 2.2-2.6%) using navigation systems [14,15]. In the present study, the occurrence of “Any deviation” in the navigation group was 8.4%, and the occurrence of deviations of more than 2 mm was 3.2%. While it cannot be claimed from the results of this study that this navigation system is superior to conventional CT-based navigation systems in improving screw insertion accuracy, it does not appear to be inferior.

In recent years, artificial intelligence (AI) has been increasingly integrated into neurosurgical procedures [16]. This navigation system includes AI-based matching of preoperative plans with the intraoperative 3D field; however, this function was not utilized in this study. While we did not evaluate this aspect, AI-assisted navigation may offer benefits beyond reducing screw deviation, such as improving screw placement accuracy according to the preoperative plan. In addition, the 3D field is recognized by a tracking system based on a different principle than conventional systems, which eliminates the need for a large, obstacle-free working space. Consequently, the surgeon does not need to make fine adjustments to their standing position and posture to operate the navigation system, potentially shortening the operative time. Although this study did not compare operative times with those observed using conventional navigation systems, there was a trend towards slightly shorter operative times than those performed under fluoroscopy (median 118 minutes vs 130 minutes). It is known that the operative time with navigation decreases with experience due to familiarity with the system [17]. Given that this study included all cases during the study period from the first case with navigation at the center under study, it is expected that the operative time will decrease further in the future. Furthermore, conventional navigation-based surgery is not expected to take less time than fluoroscopic surgery, due to the time required for setting up the system [18]. Therefore, these findings suggest that this navigation system may have the potential to reduce the operative time.

The present study suggests that this navigation system may offer superior screw insertion accuracy compared to fluoroscopic pedicle screw placement. Additionally, there is a significant advantage of the navigation-based procedure in terms of reducing radiation exposure for the surgeon [19,20]. Unfortunately, the radiation exposure for the operators in the non-navigation group (the group with screws placed under fluoroscopy) was not measured in this study; therefore, direct comparisons between groups cannot be made. However, the radiation exposure for the surgeon in the navigation group was almost zero. Recently, hand exposure within the scope of the usual practice of spine surgeons has been reported to cause chronic inflammation that shares the same origin as skin cancer [3]. The development and dissemination of techniques and surgical support equipment that reduce radiation exposure as much as possible is an urgent issue. In this respect, this compact system may contribute to their widespread use.

There are several limitations to this study. Firstly, the allocation was not randomized. Although there were no clear criteria for whether navigation was used or not, there were some differences in characteristics between the navigation and non-navigation groups, as shown in Table 1. When a new technique or device is introduced, it is often initially applied to less complex cases, while as familiarity increases, it may be used for more complex cases. Therefore, the characteristics of the navigation and non-navigation groups were likely different. We adjusted for confounding factors assumed before the study began, but unmeasured or residual confounding effects may have influenced the results as confounding by indication. Secondly, the study setting was a single center in Japan, and it is unclear whether a similar degree of preventive effect for screw deviation could be expected in other facilities. Finally, as mentioned earlier, this study only evaluated the accuracy of screw placement. The expected benefits of the new navigation system are not limited to improved screw placement accuracy; its potential to reduce the surgeon’s radiation exposure and shorten operative time should also be assessed. To claim the overall effectiveness of this navigation system, it is necessary to verify the effect of shortening the operative time and reducing the surgeon's radiation exposure. These outcomes should then be compared in a multicenter study, including centers without navigation systems and with other navigation systems.

## Conclusions

This study suggests that compact navigation systems with smart delivery tools may be associated with fewer pedicle screw deviations compared to conventional fluoroscopy-guided surgeries. However, as this was a single-center, retrospective, non-randomized study, the conclusions are limited. This study also does not include direct comparisons with other navigation systems, and claims of superior accuracy cannot be made. Notably, the system may offer unique advantages such as facilitating a better working space. These findings provide a basis for future larger, multicenter studies.
